# Art during tough times: reflections from an art-based health promotion initiative during the COVID-19 pandemic

**DOI:** 10.1177/1757975921998638

**Published:** 2021-03-25

**Authors:** Ilhan Abdullahi, Navneet Kaur Chana, Marco Zenone, Paola Ardiles

**Affiliations:** Simon Fraser University, Burnaby, British Columbia, Canada

**Keywords:** COVID-19, art, decolonization, health promotion

## Abstract

With the current COVID-19 pandemic impacting communities across the globe, diverse health promotion strategies are required to address the wide-ranging challenges we face. Art is a highly engaging tool that promotes positive well-being and increases community engagement and participation. The ‘Create Hope Mural’ campaign emerged as an arts-based health promotion response to inspire dialogue on why hope is so important for Canadians during these challenging times. This initiative is a partnership between a health promotion network based in Vancouver and an ‘open air’ art museum based in Toronto. Families were invited to submit artwork online that represents the concept of hope. This paper discusses the reflections of organizers of this arts-based health promotion initiative during the early months of the pandemic in Canada. Our findings reveal the importance of decolonizing practices, centring the voices of those impacted by crisis, while being attentive to the social and political context. These learnings can be adopted by prospective health promoters attempting to use arts-based methods to address social and health inequities.

In March 2020, the World Health Organization declared the outbreak of the novel coronavirus disease (COVID-19) to be a public health emergency of international concern (1). This pandemic has devastated the world and continues to dispropor-tionately impact underserved communities. This paper discusses the reflections of organizers of an arts-based health promotion initiative during the early months of the pandemic in Canada.

## Arts-based initiatives as a health promotion strategy

Evidence suggests that arts-based initiatives are an effective health promotion strategy and can be highly impactful in fostering community resilience and increasing the ability of communities to positively respond to traumatic events (2). In relation to this current pandemic, art can be a highly engaging tool that promotes positive well-being and increases community engagement and participation. As Springett and Masuda (3) noted, ‘participation is not just a process; it is a mindset, a philosophy of being and acting in the world ecologically, organically, and holistically, and in health promotion, with the ultimate aim of improving the conditions for optimal health and social justice.’ Arts-based approaches in health promotion can be best understood through the socio-ecological model that recognizes the collective social and environmental factors that create a distinct experience during uncertain times (4). This framework asserts that an individual’s health is in a complex, bidirectional relationship with their social environment, and thus, is directly influenced by it (5). Socio-ecological systems require approaches that are conscious of the social conditions one lives in and how these factors facilitate or challenge their health and well-being (4).

Engagement or participatory practice in health promotion is one that encourages a cyclical process of reflection and action that is designed to uncover and address the conditions undermining health and perpetuating health inequities (3). Critical voices within academia and health promotion research have called for the decolonization of health promotion strategies, which traditionally draw upon the theories of empowerment and self-efficacy (6,7). Concepts like empowerment and self-efficacy may put the onus of ill health outcomes on individuals, rather than a product of their environment and do not critically examine the history of colonialism that can inform health strategies (8). Participatory approaches focus on relational power in the process toward social change (3). Within academic literature in Canada, decolonization was a term first coined by Indigenous scholars and researchers and has increasingly become important in health and community engagement literature (8,9). Through a decolonization lens, the complex realities of individuals experiencing poorer health and mental health can be centred in health promotion initiatives through community participation.

Responses to the emerging health needs of individuals, as a result of the COVID-19 pandemic, requires the use of engaging and participatory tools that centre the experiences of communities. Therefore, this paper looks to provide insights on the learning from an interdisciplinary team of diverse members of the Create Hope campaign, an art-based health promotion initiative that emerged in Canada during the COVID-19 pandemic.

## Create Hope campaign

The Create Hope campaign emerged as a volunteer, grassroots led art-based health promotion campaign to inspire dialogue on why hope is so important during these times of the COVID-19 pandemic. The campaign launched on April 6, 2020 as a partnership between the Bridge for Health Co-operative, (on the unceded ancestral and traditional territories of the xʷməθkwəy̓əm (Musqueam), Skwxwú7mesh (Squamish), Səl̓ílwətaɬ (Tsleil-Waututh), q̓ic̓əy̓ (Katzie), and kʷikʷəƛ̓əm (Kwikwetlem) Nations) and the Dundas West Open Air Public Art Museum (on the traditional territory of many nations including the Mississaugas of the Credit, the Anishnabeg, the Chippewa, the Haudenosaunee and the Wendat peoples and is now home to many diverse First Nations, Inuit and Métis peoples. We also acknowledge that Toronto is covered by Treaty 13 with the Mississaugas of the Credit), (10–12). Bridge for Health is a co-operative in Metro Vancouver that utilizes a community-based model to promote well-being and equity (10). The Dundas West Open Air Museum is an artist and community driven initiative that builds on mural art and shares the art and history of the diverse communities in the DundasWest neighbourhood in Toronto (11). By working in partnership, the campaign’s goal was to find a way to encourage dialogue on hope during the COVID-19 pandemic.

**Figure 1.**
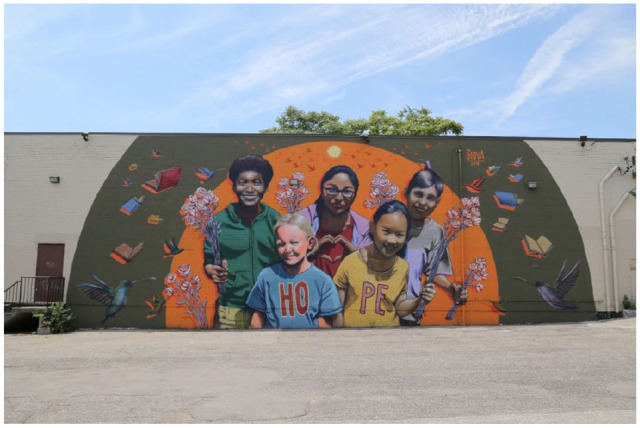
Mockup image of the hope mural by Giovanni Zamora.

The campaign organizers included a dedicated group of volunteers comprised of health promoters, public health professionals, community organizers, local artists, social entrepreneurs, and university students, of varying ages, abilities, gender and racial identities. The campaign consisted of four phases: Phase I: invitation to submit artwork on Bridge for Health social media platforms (Twitter, Facebook, Instagram) and through the Bridge for Health website (www.bridgeforhealth.org). Phase II: publication of artwork submitted to the campaign on Bridge for Health’s website and social media channels using @Bridge4Health and hashtag #CreateHopeMural. Phase III: The design of a digital collage which captures all of the images collected into one digital collage with the word ‘hope’ as the backdrop. Phase IV: A physical mural to be painted on the streets of Toronto, in the Dundas West museum neighbourhood once the pandemic restrictions are lifted (Figure 1). In a 6-week period, from April to May 2020, we collected a total of 30 art pieces; the majority of submissions were from family members and individuals who primarily resided in Metro Vancouver and the Greater Toronto area, between the ages of 5 and 18 years (with prior consent).The following section will discuss some of the reflections and learnings that resulted from the Create Hope campaign.

## Reflections from campaign organizers

### Health is political

What emerged as a response to provide hope during the current COVID-19 pandemic resulted in a deeply reflective process for the campaign organizers. Adaptability and resiliency surfaced as key themes and strategies for the organizers to navigate the complex realities of living through a pandemic. The COVID-19 pandemic has proven to be a difficult time for families, communities and professionals working to address its implications. At a time when uncertainty is heightened by a pandemic, the world is simultaneously witnessing a global movement to address police brutality, anti-Black violence and racism experienced by Black communities in the United States and echoed worldwide. In the early months of summer 2020, Black Lives Matter (BLM) protests took place across the globe, making it a pivotal and monumental moment in history. Moreover, the pandemic also made visible in the public eye, the brutality of how Indigenous peoples are treated in the health-care system across Canada. After an in-depth investigation, a report was released in November documenting racism, stereotyping and discrimination against Indigenous peoples in British Columbia (13). These events clearly illustrated the interconnectedness of social and health systems within society and their ramifications on the health of underserved communities.

For the Create Hope organizers, the calls for anti-racism work sparked conversations on how creating hope for the post-pandemic era necessarily required an examination of the colonial past and histories as a fundamental cause of health inequities and reinforced the notion that health is political. That is, it is necessary to conceptualize new possible outcomes as products of socio-political circumstances, while understanding the complex, often intersecting social and health systems (14).

Health promoters, public health professionals and community organizers cannot be divorced from the complex realities of our society. For health promotion interventions to be effective, they need to centre the experiences and voices of those impacted while being responsive to current events. As noted by Boutilier and Mason, health promotion practitioners can participate in collaborative reflection, leading to ongoing questioning of the complex and dynamic nature of practice, while paying attention to the process itself and issues that contribute to building trust among practitioners (15).

A critical learning for campaign organizers was to be attentive to the social context and to be responsive to current events and establish interventions that can be adaptable. Organizers had to shift their language, social media messages and thinking along the way, in order to respond to the context that was constantly changing at the height of the start of the pandemic. One of the key insights gained along the way was the need to understand their own social location as organizers and how the various identities, agencies and experiences of the team enriched (and challenged) the experience of organizing. The organizers had not anticipated at the start that they would need a process to keep track of their dialogue and reflection. It was soon realized that for any kind of evaluation of their work in the future, they needed to have a mechanism to capture the dynamic decision making and thought processes that impacted how they continuously tailored and adapted their engagement strategy.

### Health promotion campaigns need a decolonizing approach

The concept of decolonization ended up becoming a key principle of this campaign and continues to inform future directions of the Create Hope campaign. It was practised by questioning the colonial histories, as well as the dominant western ways of ‘knowing’ and ‘doing’. Organizers had an opportunity to create their own understanding of how their experiences shaped their knowledge and vice-versa. Importantly, it meant working alongside communities impacted by the issue at hand and building meaningful and mutual relationships with diverse communities while understanding the colonial histories underpinning their social conditions (16). For the organizers leading the Create Hope campaign, critical reflexiveness on their social location and their own healing journeys was integral in informing the design and direction of this campaign. Their healing journeys centred around the importance of ‘hope’ itself, as well as nature, staying connected to the land and using art in their own coping and resilience strategies. In essence, the campaign unexpectedly served as a healing journey for the organizers.

## Conclusion

This paper provides insights into critical elements of developing a health promotion campaign, centred on the reflexiveness of health promoters and campaign organizers, to support communities with dialogue around hope during the COVID-19 pandemic. It is hoped that the experiences shared by campaign organizers in developing this health promotion initiative will highlight the importance of reflective practice, as well as the dynamic nature of health promotion requiring adaptable and creative strategies that centre individuals and communities, and their expressions during a time of crisis.
